# Supplementary material for: Extreme diversity of phage amplification rates and phage–antibiotic interactions revealed by PHORCE
Source: PLoS Biol. 2025 Apr 8;23(4):e3003065. doi: 10.1371/journal.pbio.3003065 (PMC12013923; doi:10.1371/journal.pbio.3003065)
Supplement: S1 Text — (PDF) [file pbio.3003065.s001.pdf]

S1 Text. Requirements to determine the phage amplification rate

To ease the process of implementing PHORCE, we here summarize the requirements to determine the phage amplification rate. The phage amplification rate can be measured from a single growth curve, following Eq. 10. One needs to measure the following quantities:

Collapse time (t_{col}): one can extract this from phage predation assay. It is important that the bacteria follow steady state growth throughout the entire experiment. Therefore, one needs to preculture the bacterial population in the relevant medium (e.g. incl. antibiotics) for at least 5 cell divisions before adding phage. The time point at which the phage is added is taken as time zero.

Bacterial growth rate r_{bac}: The bacterial growth rate can be extracted from the same curve as the collapse time. However, the curve is relatively short for phages with an early collapse time. Therefore, for each condition we measure the growth curves with phages to extract the collapse time and without phage to extract the bacterial growth rate.

Initial bacterial concentration b_0: There are two ways to estimate the initial bacterial concentration: either by quantitatively measuring the concentration or by semi-quantitatively estimating it from the growth curve. Both are detailed below.

Option 1: To quantitatively measure the bacterial concentration, a sample needs to be taken from the bacterial preculture just before adding phages. From this sample, the bacterial concentration can be measured using colony counting, a coulter counter, flow cytometry, or similar.

Option 2: The initial bacterial concentration can be semi-quantitatively estimated from the bacterial growth curves by extrapolating the bacterial growth curve (either optical density or bioluminescence) to time zero. This value can then be used as a semi-quantitative proxy for initial bacterial density. Using this approach, different phages can be compared semi-quantitatively with each other within the same growth conditions. Between growth conditions, the relationship between optical density (or especially bioluminescence) and bacterial density becomes unclear and therefore requires verification per condition (e.g., S9c Fig).

Ratio between the final and initial phage concentration p_{inf}/p_0: Lastly, there are three options to estimate the ratio between the final and initial phage concentrations: with plaque assay, via growth curves or by assuming the stock concentration of phages is the same as the phage concentration at the end.

Option 1: One can quantitatively measure the phage concentration of both the initial and final phage concentration using the plaque assay.

Option 2: One can use bacterial growth curves to estimate phage concentrations ([9] and S2 Fig). There is no need to quantify the stock concentration using the plaque assay as only the ratio, and not the absolute numbers, of the final and initial phage concentration (p_{inf}/p_0) is required.

Option 3: We noticed that the final phage concentration is typically close to the phage stock concentration. Furthermore, the phage amplification rate only weakly depends on the ratio between the final and initial phage concentration. Therefore, one can approximate the ratio $p_{\mathrm{inf}}/p_0 \approx p_{\mathrm{stock}}/p_0$, which is the dilution factor of the stock solution when inoculating the bacterial sample. Note that this approximation likely works best when the phage stock solution was made under similar conditions (bacterial host, culturing conditions etc.) as the conditions in which the phage amplification rate is assessed.
