# Supplementary material for: Extreme diversity of phage amplification rates and phage–antibiotic interactions revealed by PHORCE
Source: PLoS Biol. 2025 Apr 8;23(4):e3003065. doi: 10.1371/journal.pbio.3003065 (PMC12013923; doi:10.1371/journal.pbio.3003065)
Supplement: S1 Fig — (a) Example of an E. coli growth curve in the absence (blue) and presence (orange) of a phage (Bas04), as measured by optical density (Methods). Initially, there was condensation on the lid of the 96-well plate, resulting in an artifactually higher optical density for the first half hour. (b) The collapse time of phage Bas04 measured with both bioluminescence and optical density for the same samples. All combinations of seven MOIs (0.0004–0.3, 3× steps) and 11 bacterial concentrations (3 × 10^3^–3 × 10^6^, 2× steps) were measured. Both methods show close agreement (Pearson’s ρ = 0.99, p = 10−65). The data underlying this figure can be found in S1 Data. (PDF) [file pbio.3003065.s002.pdf]

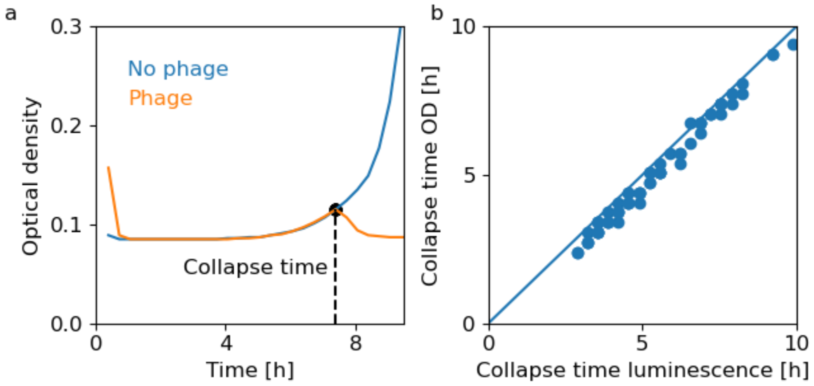

S1 Fig. Comparison of collapse times obtained from bioluminescence and optical density measurements. a) Example of an *E. coli* growth curve in the absence (blue) and presence (orange) of a phage (Bas04), as measured by optical density (Methods). Initially there was condensation on the lid of the 96-well plate, resulting in an artifactually higher optical density for the first half hour. **b)** The collapse time of phage Bas04 measured with both bioluminescence and optical density for the same samples. All combinations of seven MOIs (0.0004 – 0.3, 3× steps) and 11 bacterial concentrations (3×10^3 – 3×10^6, 2× steps) were measured. Both methods show close agreement (Pearson's ρ=0.99, p=10^{-65}). The data underlying this Figure can be found in S1 Data.
