# Supplementary material for: Extreme diversity of phage amplification rates and phage–antibiotic interactions revealed by PHORCE
Source: PLoS Biol. 2025 Apr 8;23(4):e3003065. doi: 10.1371/journal.pbio.3003065 (PMC12013923; doi:10.1371/journal.pbio.3003065)
Supplement: S2 Fig — (a) Bacterial growth curves were measured in the presence of several different dilutions (1/800, 1/1,600, and 1/3,200, respectively, orange, pink, and blue) of the Bas04 phage stock used in Figs 1 and 2 at a fixed initial bacterial concentration (4 × 105 mL−1) to quantify the phage concentration-dependent collapse time. (b) We fit these collapse times (tcol) with a logarithmic decay (black line, see Ref. [9] for details) to obtain a calibration curve that we use to determine unknown phage concentrations in samples containing the phage Bas04. The data underlying this figure can be found in S1 Data. (PDF) [file pbio.3003065.s003.pdf]

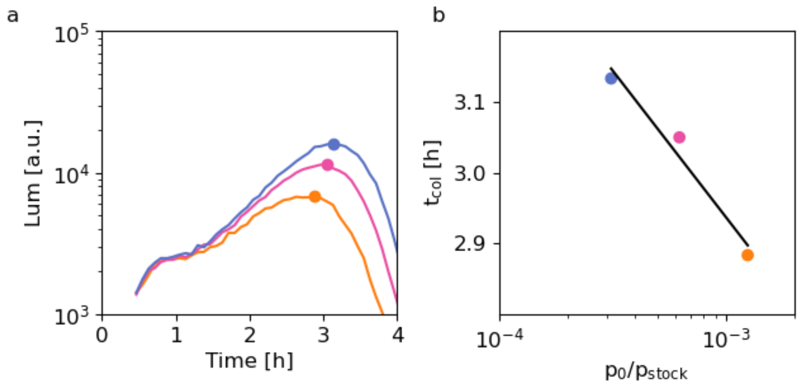

S2 Fig. Calculation of the phage concentration from growth curves. a) Bacterial growth curves were measured in the presence of several different dilutions (1/800, 1/1600 and 1/3200, respectively orange, pink and blue) of the Bas04 phage stock used in Figs. 1 and 2 at a fixed initial bacterial concentration (4×10^5 ml^{-1}) to quantify the phage concentration-dependent collapse time. **b)** We fit these collapse times (t_{col}) with a logarithmic decay (black line, see [9] for details) to obtain a calibration curve that we use to determine unknown phage concentrations in samples containing the phage Bas04. The data underlying this Figure can be found in S1 Data.
