# Supplementary material for: Extreme diversity of phage amplification rates and phage–antibiotic interactions revealed by PHORCE
Source: PLoS Biol. 2025 Apr 8;23(4):e3003065. doi: 10.1371/journal.pbio.3003065 (PMC12013923; doi:10.1371/journal.pbio.3003065)
Supplement: S3 Fig — Comparison between measured bacterial growth curves (black) and model trajectories (red, dotted) as a function of the different initial bacterial concentrations (1.1 × 10^4^–1.1 × 106 mL−1, left to right) and 10 different initial phage concentrations (2.5 × 10^3^–1.3 × 106 mL−1, top to bottom). The procedure for calculating the model trajectories is explained in section Comparison of full growth curves between model and experiments. Overall, the model trajectories agree well with the measured bacterial growth curves up to collapse and capture the collapse time. The quality of the fit depends on the initial conditions: The higher the initial bacterial concentration, the further the system is from the adsorption-limited regime, and the worse the agreement with the model. The experimental data underlying this figure can be found in S1 Data. (PDF) [file pbio.3003065.s004.pdf]

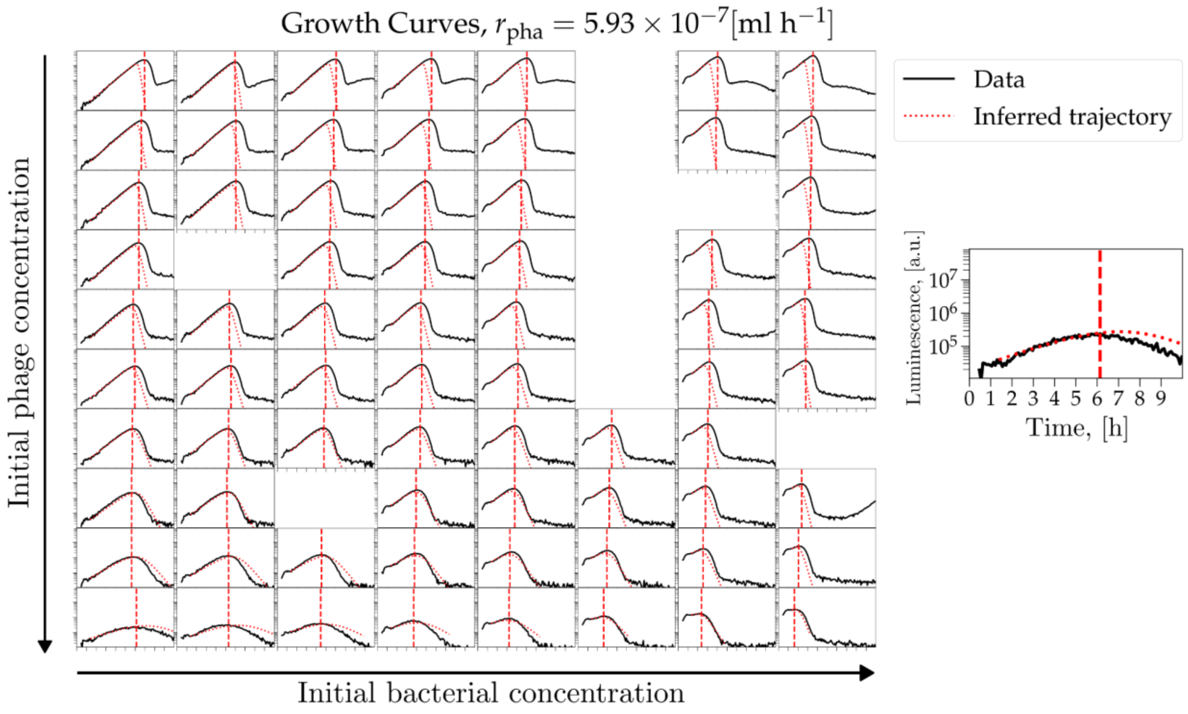

S3 Fig. Comparison of full growth curves between model and experiments. Comparison between measured bacterial growth curves (black) and model trajectories (red, dotted) as a function of the different initial bacterial concentrations (1.1×10^4 – 1.1×10^6 ml^{-1}, left to right) and ten different initial phage concentrations (2.5×10^3 – 1.3×10^6 ml^{-1}, top to bottom). The procedure for calculating the model trajectories is explained in section *Comparison of full growth curves between model and experiments*. Overall, the model trajectories agree well with the measured bacterial growth curves up to collapse and capture the collapse time. The quality of the fit depends on the initial conditions: the higher the initial bacterial concentration, the further the system is from the adsorption-limited regime, and the worse the agreement with the model. The experimental data underlying this Figure can be found in S1 Data.
