# Supplementary material for: Extreme diversity of phage amplification rates and phage–antibiotic interactions revealed by PHORCE
Source: PLoS Biol. 2025 Apr 8;23(4):e3003065. doi: 10.1371/journal.pbio.3003065 (PMC12013923; doi:10.1371/journal.pbio.3003065)
Supplement: S4 Fig — Results of a mathematical model including phage resistance and lysogeny (Eqs. 17−20). When resistance (orange dashed line) and lysogeny (green dashed line) are included, the dynamics up to the collapse time and including the initial collapse are not noticeably affected. The curve for resistance and lysogeny is not shown because it is indistinguishable from the curve for lysogeny alone. For the simulations, we used an initial bacterial density of 105cfu/mL, an initial phage density of 105 pfu/mL, a bacterial growth rate of 1 h−1, a resistance frequency of 10−8, an adsorption rate of 10−9 cfu/mL/h, a burst size of 100, and a lysogeny frequency of 10−6, which are all realistic parameters [9]. The y-axis shows the total bacterial population (sum of the sensitive, resistant, and lysogenized populations). (PDF) [file pbio.3003065.s005.pdf]

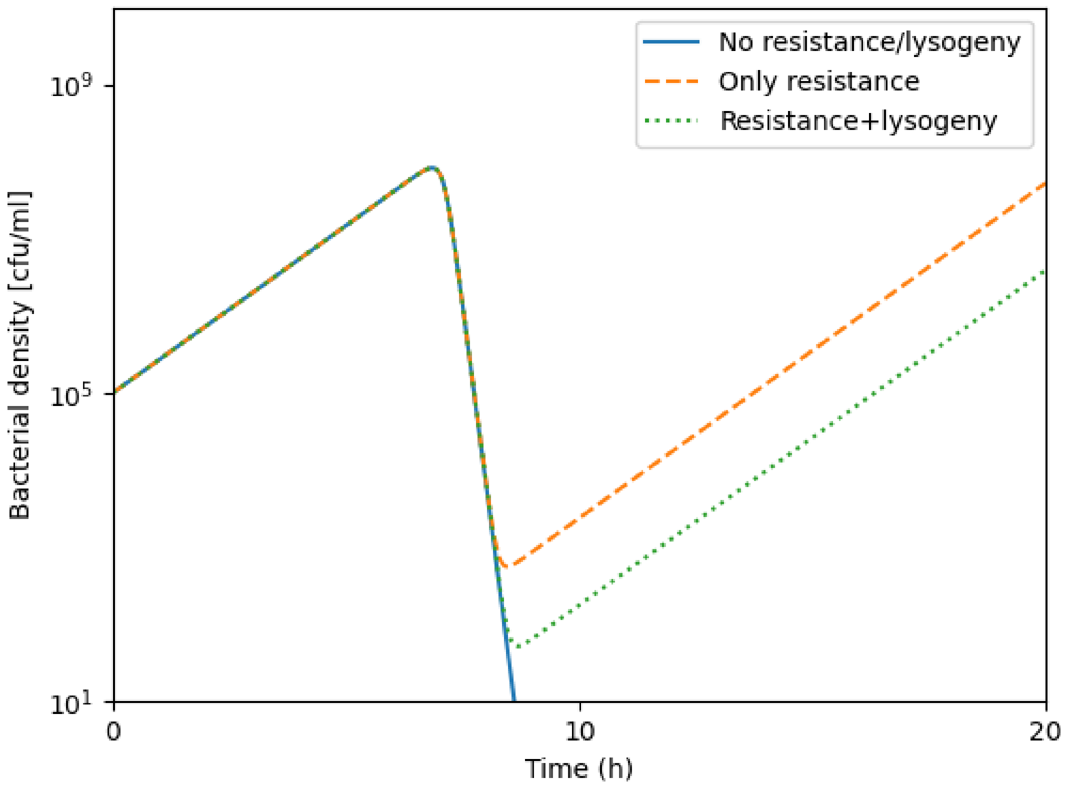

S4 Fig. Resistance and lysogeny affect only post-collapse dynamics. Results of a mathematical model including phage resistance and lysogeny (Eqs. 17-20). When resistance (orange dashed line) and lysogeny (green dashed line) are included, the dynamics up to the collapse time and including the initial collapse are not noticeably affected. The curve for resistance and lysogeny is not shown because it is indistinguishable from the curve for lysogeny alone. For the simulations, we used an initial bacterial density of 10^5 cfu/ml, an initial phage density of 10^5 pfu/ml, a bacterial growth rate of 1 h^{-1}, a resistance frequency of 10^{-8}, an adsorption rate of 10^{-9} cfu/ml/h, a burst size of 100, and a lysogeny frequency of 10^{-6}, which are all realistic parameters [9]. The y-axis shows the total bacterial population (sum of the sensitive, resistant, and lysogenized populations).
