# Supplementary material for: Extreme diversity of phage amplification rates and phage–antibiotic interactions revealed by PHORCE
Source: PLoS Biol. 2025 Apr 8;23(4):e3003065. doi: 10.1371/journal.pbio.3003065 (PMC12013923; doi:10.1371/journal.pbio.3003065)
Supplement: S5 Fig — We measured growth curves of a P. aeruginosa clinical isolate in the presence of a therapeutically used phage (Methods) to test whether PHORCE also works for bacteria other than E. coli laboratory strains. (a) Bacterial growth curves from a two-dimensional gradient of initial bacterial densities (serially diluted by a factor of 3, from left to right: 460–3.3 × 10^5^ cfu/mL) and initial phage densities (serially diluted by a factor of 3, from black to red: 30–1.7 × 10^6^ pfu/mL). (b) Collapse time versus initial bacterial density for the experiment in a. Similar to our results for E. coli (Fig 1c), the collapse time decreases logarithmically with the bacterial density for a fixed initial bacterial-to-phage ratio (CFU/PFU = 0.2). (c) The PHORCE model (Eq 1) quantitatively captures the dependence of the collapse time on the initial bacterial and phage densities, using the phage amplification rate as the only free parameter. The fitted phage amplification rate was 1.4 × 10−6 mL h−1, which is comparable to the strongest BASEL phage. Such a high rate is plausible because this phage was selected for its strong activity. The data underlying this figure can be found in S1 Data. (PDF) [file pbio.3003065.s006.pdf]

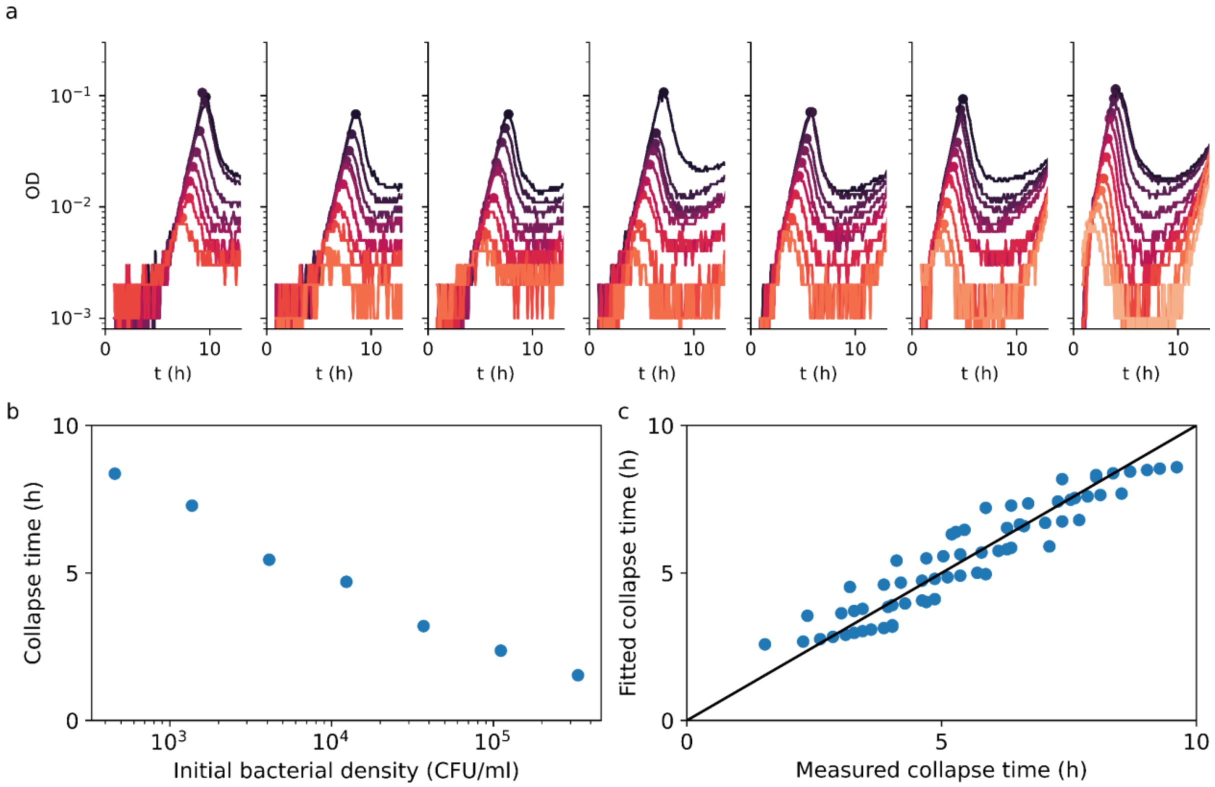

S5 Fig. PHORCE also captures the collapse time for a *Pseudomonas aeruginosa* strain and its phage. We measured growth curves of a *Pseudomonas aeruginosa* clinical isolate in the presence of a therapeutically used phage (Methods) to test whether PHORCE also works for bacteria other than *E. coli* laboratory strains. **a)** Bacterial growth curves from a two-dimensional gradient of initial bacterial densities (serially diluted by a factor of 3, from left to right: 460 - 3.3×10^5 cfu/ml) and initial phage densities (serially diluted by a factor of 3, from black to red: 30 - 1.7×10^6 pfu/ml). **b)** Collapse time versus initial bacterial density for the experiment in **a**. Similar to our results for *E. coli* (Fig. 1c), the collapse time decreases logarithmically with the bacterial density for a fixed initial bacterial-to-phage ratio (CFU/PFU = 0.2). **c)** The PHORCE model (Eq. 1) quantitatively captures the dependence of the collapse time on the initial bacterial and phage densities, using the phage amplification rate as the only free parameter. The fitted phage amplification rate was 1.4×10^{-6} ml h^{-1}, which is comparable to the strongest BASEL phage. Such a high rate is plausible because this phage was selected for its strong activity. The data underlying this Figure can be found in S1 Data.
