# Supplementary material for: Extreme diversity of phage amplification rates and phage–antibiotic interactions revealed by PHORCE
Source: PLoS Biol. 2025 Apr 8;23(4):e3003065. doi: 10.1371/journal.pbio.3003065 (PMC12013923; doi:10.1371/journal.pbio.3003065)
Supplement: S6 Fig — Each graph shows a time-resolved bacterial growth curve, measured using bioluminescence (Methods), in the presence of a different phage. Green to black shows different initial bacterial concentrations (resp. 480, 1.44 × 10^4^ and 4.32 × 105 mL−1) at a fixed initial MOI, and red to black shows different phage concentrations at a fixed initial bacterial concentration (resp. 8.1 × 10^5^, 2.7 × 10^4^ and 900× diluted from the phage stock solutions). The first column shows the BASEL phages 1–12, the second column shows 13–24, and so on (top to bottom). The data underlying this figure can be found in S1 Data. (PDF) [file pbio.3003065.s007.pdf]

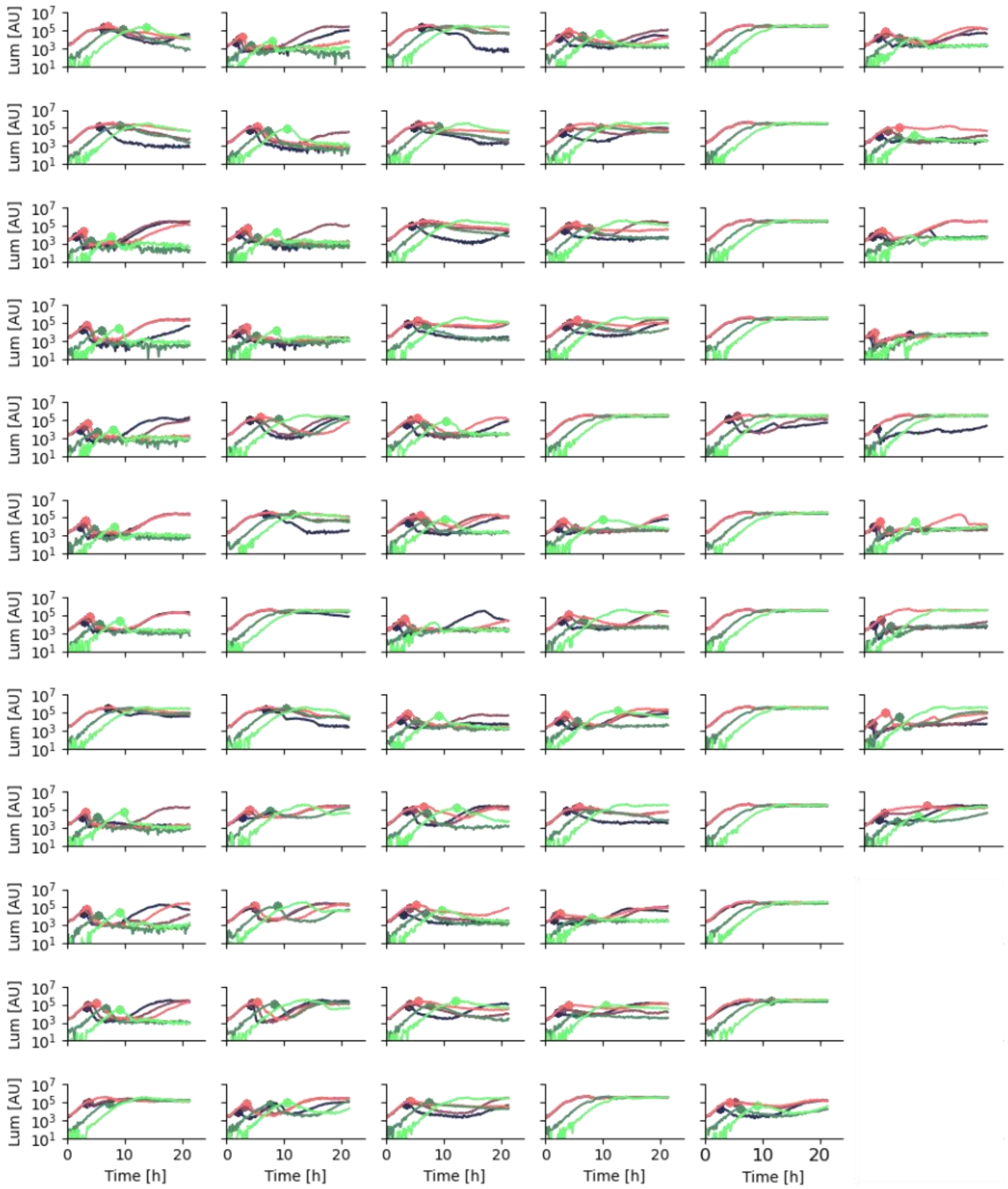

S6 Fig. Raw bacterial growth curves in the presence of the BASEL phages. Each graph shows a time-resolved bacterial growth curve, measured using bioluminescence (Methods), in the presence of a different phage. Green to black shows different initial bacterial concentrations (resp. 480, 1.44×10^4 and 4.32×10^5 ml^{-1}) at a fixed initial MOI and red to black shows different phage concentrations at a fixed initial bacterial concentration (resp. 8.1×10^5, 2.7×10^4 and 900× diluted from the phage stock solutions). The first column shows the BASEL phages 1–12, the second column shows 13–24, and so on (top to bottom). The data underlying this Figure can be found in S1 Data.
