# Supplementary material for: Extreme diversity of phage amplification rates and phage–antibiotic interactions revealed by PHORCE
Source: PLoS Biol. 2025 Apr 8;23(4):e3003065. doi: 10.1371/journal.pbio.3003065 (PMC12013923; doi:10.1371/journal.pbio.3003065)
Supplement: S7 Fig — Analysis of the growth curves in S6 Fig. (a) The collapse time was measured at three bacterial densities, keeping the ratio of phages to bacteria (multiplicity of infection [MOI]) fixed, for the entire BASEL collection. tcol,1−3 indicate time of collapse at resp. the lowest, middle and highest bacterial density. Each data point represents a different phage. All data points below the two solid lines show a monotonic increase in collapse time as a function of the bacterial concentration. The dashed line indicates where the collapse time increases with the logarithm of the bacterial concentration. The vast majority of data points lie below the two solid lines and cluster around the dashed line, suggesting that the amplification kinetics of all BASEL phages are predominantly adsorption-limited. (b) Phage amplification rates for different phage morphotypes, 3/6 podoviruses, 0/34 siphoviruses, and 8/29 myoviruses had a phage amplification rate below the detection limit (3 × 10−10 mL h−1). None of the morphotypes differ significantly from the others (p = 0.08, Kruskal–Wallis) and any differences become even more insignificant if the vequintavirus members (Fig 3e) are excluded from the myoviruses (p = 0.87, Kruskal–Wallis). (c) Comparison between the overnight yield (y-axis) and the phage amplification rate (x-axis) for each phage in the BASEL collection shows no significant correlation (τ = −0.04, p = 0.6, Kendall rank correlation). (d) Comparison between the phage amplification rate (x-axis) and its approximation by assuming p∞/p0=pstock/p0 (y-axis). Each data point shows the phage amplification rate determined from a single amplification curve (nine phages, three replicates per condition). Both results are in close agreement (Pearson’s ρ = 0.95, p = 10−22). The data underlying this figure can be found in S1 Data. (PDF) [file pbio.3003065.s008.pdf]

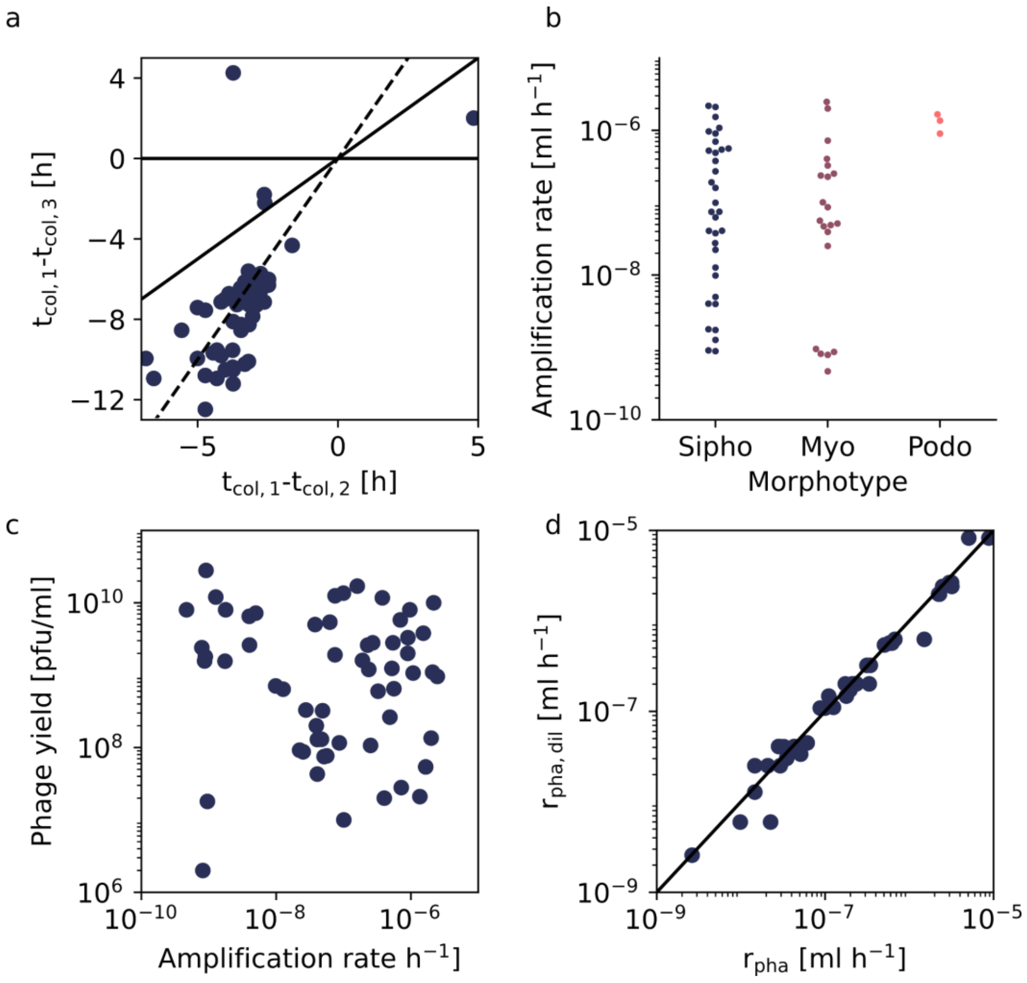

S7 Fig. Phage amplification characteristics of the BASEL collection. Analysis of the growth curves in S6 Fig. **a)** The collapse time was measured at three bacterial densities, keeping the ratio of phages to bacteria (multiplicity of infection, MOI) fixed, for the entire BASEL collection. $t_{\mathrm{col},1-3}$ indicate time of collapse at resp. the lowest, middle and highest bacterial density. Each data point represents a different phage. All data points below the two solid lines show a monotonic increase in collapse time as a function of the bacterial concentration. The dashed line indicates where the collapse time increases with the logarithm of the bacterial concentration. The vast majority of data points lie below the two solid lines and cluster around the dashed line, suggesting that the amplification kinetics of all BASEL phages are predominantly adsorption-limited. **b)** Phage amplification rates for different phage morphotypes, 3/6 podoviruses, 0/34 siphoviruses and 8/29 myoviruses had a phage amplification rate below the detection limit (3×10^{-10} ml h^{-1}). None of the morphotypes differ significantly from the others (*p*=0.08, Kruskal-Wallis) and any differences become even more insignificant if the vequintavirus members (Fig. 3e) are excluded from the myoviruses (*p*=0.87, Kruskal-Wallis). **c)** Comparison between the overnight yield (y-axis) and the phage amplification rate (x-axis) for each phage in the BASEL collection shows no significant correlation (τ=-0.04, p=0.6, Kendall rank correlation) **d)** Comparison between the phage amplification rate (x-axis) and its approximation by assuming $p_\infty/p_0 = p_{\mathrm{stock}}/p_0$ (y-axis). Each data point shows the phage amplification rate determined from a single amplification curve (nine phages, three replicates per condition). Both results are in close agreement (Pearson's ρ=0.95, $p=10^{-22}$). The data underlying this Figure can be found in S1 Data.
