# Supplementary material for: Extreme diversity of phage amplification rates and phage–antibiotic interactions revealed by PHORCE
Source: PLoS Biol. 2025 Apr 8;23(4):e3003065. doi: 10.1371/journal.pbio.3003065 (PMC12013923; doi:10.1371/journal.pbio.3003065)
Supplement: S8 Fig — Bioluminescence as a function of time. Each row shows a different phage from the BASEL collection (phages from top to bottom: Bas01, Bas04, Bas10, Bas14. Bas17, Bas23, Bas30, Bas37, Bas42, Bas55, and Bas63); each column shows a different antibiotic (left: nitrofurantoin, orange; right, doxycycline, blue) at different concentrations (0.02–0.2 × MIC from dark to colored, where black is no antibiotic). The data underlying this figure can be found in S1 Data. (PDF) [file pbio.3003065.s009.pdf]

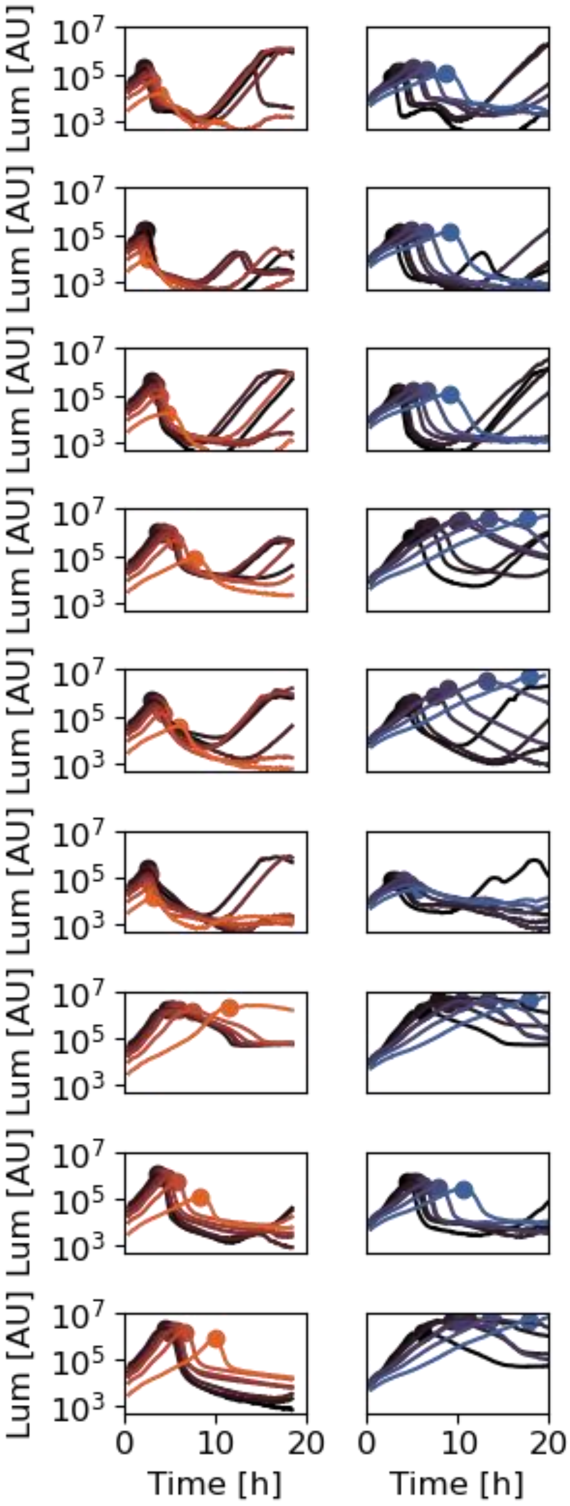

S8 Fig. Bacterial growth curves in the presence of antibiotics and phages. Bioluminescence as a function of time. Each row shows a different phage from the BASEL collection (phages from top to bottom: Bas01, Bas04, Bas10, Bas14. Bas17, Bas23, Bas30, Bas37, Bas42, Bas55, Bas63), each column shows a different antibiotic (left: nitrofurantoin, orange; right, doxycycline, blue) at different concentrations (0.02 - 0.2 × MIC from dark to colored, where black is no antibiotic). The data underlying this Figure can be found in S1 Data.
