# Supplementary material for: Extreme diversity of phage amplification rates and phage–antibiotic interactions revealed by PHORCE
Source: PLoS Biol. 2025 Apr 8;23(4):e3003065. doi: 10.1371/journal.pbio.3003065 (PMC12013923; doi:10.1371/journal.pbio.3003065)
Supplement: S9 Fig — (a) The degree of correlation between the phage amplification rate and the antibiotic concentration was assessed using either a Kendall correlation test (x-axis) or a Pearson correlation test (y-axis). Both scores are significantly correlated with each other (Kendall’s τ = 0.5, p = 0.002). (b) No correlation was found between the interaction score based on the phage yield and the interaction score based on the amplification rate (p = 0.6, Kendall rank correlation). (c) The phage amplification rate was calculated either by explicitly measuring the initial bacterial concentration and the final phage concentration (x-axis) or by approximating the initial bacterial concentration with the initial bioluminescence value and assuming p∞/p0=pstock/p0 (y-axis) (Methods). The explicit and approximate approaches yield similar results (Pearson’s ρ = 0.67, p = 10−44). On both axes, the antibiotic-dependent phage amplification rate was normalized to the phage amplification rate in the absence of the drug. For both doxycycline and nitrofurantoin, nine phages and eight drug concentrations were measured, respectively. The data underlying this figure can be found in S1 Data. (PDF) [file pbio.3003065.s010.pdf]

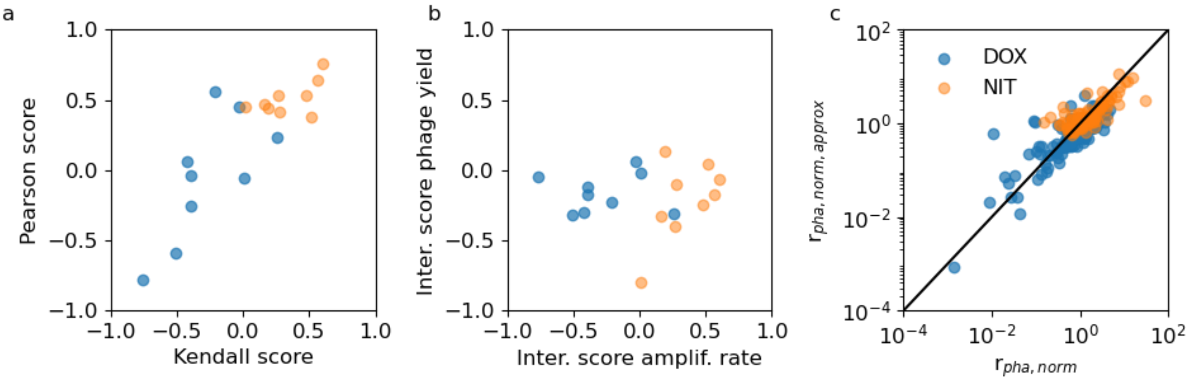

S9 Fig. Phage-antibiotic interactions. a) The degree of correlation between the phage amplification rate and the antibiotic concentration was assessed using either a Kendall correlation test (x-axis) or a Pearson correlation test (y-axis). Both scores are significantly correlated with each other (Kendall's τ =0.5, p=0.002). **b)** No correlation was found between the interaction score based on the phage yield and the interaction score based on the amplification rate (p=0.6, Kendall rank correlation). **c)** The phage amplification rate was calculated either by explicitly measuring the initial bacterial concentration and the final phage concentration (x-axis) or by approximating the initial bacterial concentration with the initial bioluminescence value and assuming $p_\infty/p_0 = p_{\text{stock}}/p_0$ (y-axis) (Methods). The explicit and approximate approaches yield similar results (Pearson's ρ=0.67, $p=10^{-44}$). On both axes, the antibiotic-dependent phage amplification rate was normalized to the phage amplification rate in the absence of the drug. For both doxycycline and nitrofurantoin, nine phages and eight drug concentrations were measured, respectively. The data underlying this Figure can be found in S1 Data.
