# Supplementary material for: Extreme diversity of phage amplification rates and phage–antibiotic interactions revealed by PHORCE
Source: PLoS Biol. 2025 Apr 8;23(4):e3003065. doi: 10.1371/journal.pbio.3003065 (PMC12013923; doi:10.1371/journal.pbio.3003065)
Supplement: S10 Fig — Results of an experiment with a two-dimensional gradient of initial phage (Bas14) and bacterial concentration, similar to Fig 2. (a) Bacterial growth curves in the two-dimensional gradient. Different columns show different initial bacterial densities (left to right: 1:100–1:12,800 dilutions from a 1:100 diluted overnight culture pre-cultured for 2 h using seven steps of 5-fold serial dilutions); different colors show different initial phage concentrations (black to red: 0.001–6.25 × 10^−5^ relative to the stock concentration using four steps of 5-fold serial dilutions). (b) Phage yield versus collapse time. We took phage samples at t = 8 h (blue) and at t = 21 h (orange) and measured the relative phage concentration by reinoculation (S2 Fig). At these time points, the bioluminescence drops slightly as the cultures are removed from the plate reader and therefore cool down slightly before being returned to the temperature-controlled plate reader. Since the samples have different initial conditions, these yields (blue data points) correspond to different time points relative to the collapse time: The x-axis shows the time of the first phage concentration measurement (t = 8 h) minus the collapse time. (c) Ratio of phage amplification rate calculated from the phage yields measured after 8 h and after 21 h. The data underlying this figure can be found in S1 Data. (PDF) [file pbio.3003065.s011.pdf]

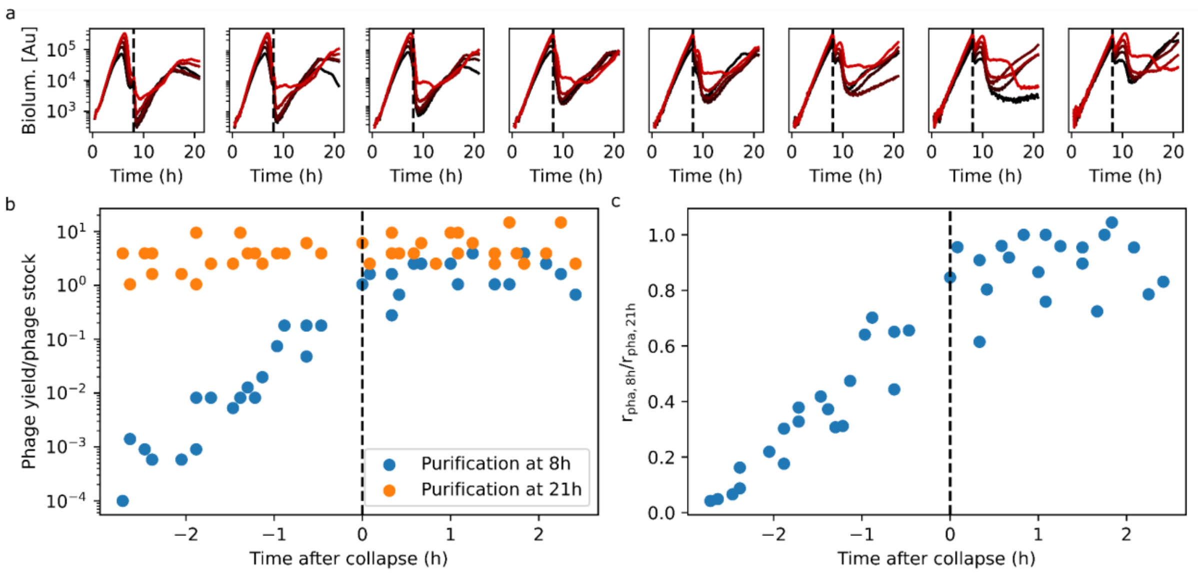

S10 Fig. Dependence of phage yield on time of measurement. Results of an experiment with a two-dimensional gradient of initial phage (Bas14) and bacterial concentration, similar to Fig. 2. **a)** Bacterial growth curves in the two-dimensional gradient. Different columns show different initial bacterial densities (left to right: 1:100 - 1:12 800 dilutions from a 1:100 diluted overnight culture pre-cultured for 2 h using 7 steps of 5-fold serial dilutions); different colors show different initial phage concentrations (black to red: 0.001 - 6.25×10^{-5} relative to the stock concentration using 4 steps of 5-fold serial dilutions). **b)** Phage yield versus collapse time. We took phage samples at t = 8 h (blue) and at t = 21 h (orange) and measured the relative phage concentration by reinoculation (S2 Fig). At these time points, the bioluminescence drops slightly as the cultures are removed from the plate reader and therefore cool down slightly before being returned to the temperature-controlled plate reader. Since the samples have different initial conditions, these yields (blue data points) correspond to different time points relative to the collapse time: the x-axis shows the time of the first phage concentration measurement (t = 8 h) minus the collapse time. **c)** Ratio of phage amplification rate calculated from the phage yields measured after 8 h and after 21 h. The data underlying this Figure can be found in S1 Data.
