# Supplementary material for: Extreme diversity of phage amplification rates and phage–antibiotic interactions revealed by PHORCE
Source: PLoS Biol. 2025 Apr 8;23(4):e3003065. doi: 10.1371/journal.pbio.3003065 (PMC12013923; doi:10.1371/journal.pbio.3003065)
Supplement: Attachment — Submitted filename: PHORCE_point_by_point_response.pdf [file pbio.3003065.s016.pdf]

Point-by-point response

Reviewer #1:

Mulla et al. present an exciting new method to measure phage amplification rates in a high-throughput manner. They show that phage infection kinetics under assay conditions seem to be adsorption-limited and derive an analytical expression for the collapse time of the bacterial population. They use their method to show the wide diversity of phage amplification rates, which do not correlate with phage morphology. They also find that interactions between phages and antibiotic are determined largely by the antibiotic and not the phage species. Overall, the paper presents a valuable method that could alleviate a current limitation in phage research. The authors underline this by presenting novel insights into phage amplification and phage-antibiotic interactions. My comments mainly concern unclarities about the method and presentation of its output.

1) Model: How dependent is the model on an accurate estimate of bacterial growth rate? At least for optical density measurements, growth rate estimates might be affected by changes in cell size, e.g. through filamentation in the presence of antibiotics or between different media conditions.

We thank the reviewer for pointing this out. The phage amplification rate depends exponentially on the bacterial growth rate, which is indeed quite sensitive. One of the reasons our approach still works is that changes in phage amplification rate tend to be quite large across different phages and antibiotics. Transient phenomena such as filamentation are generally a problem for accurate measurements of bacterial growth rates. However, this problem can be largely avoided by ensuring that the bacterial culture is in a steady state of exponential growth. To ensure steady state growth, we use pre-cultures in the same media as in which we test our phage activity: we inoculate bacteria for several (typically 5-10) cell divisions before adding phages, which is particularly important in combination with antibiotics. In steady state growth, transient phenomena such as filamentation or changes in cell size are no longer an issue. Also, when comparing different phages for the same condition, this is less of an issue, as the relative changes in phage amplification rate are easily detected, even if the measured absolute bacterial growth rate is slightly imprecise.

We have added a more detailed explanation of this in the main text (lines 222-224) and in the Supplementary Information (lines 847-850).

2) Plate preparation: the dilutions are described in a relatively convoluted way in the section 'Two-dimensional gradient of initial phage and bacteria concentration', which does not particularly lend itself to repeating the method. It is also unclear how the double gradient was achieved as it sounds like first the phage gradient was pipetted and then the bacterial gradient was also done by dilution. Would that not change the phage concentration in the wells and how was this calculated?
It would be helpful to have a schematic of how the experiments were performed to make reproduction and understanding easier.

Thank you for pointing out this issue, which was also mentioned by reviewer #3 below. We agree that the previous description of the dilution was difficult to understand. Regarding the specific question: It is indeed necessary to take into account the dilution by the second gradient when setting up the first gradient. The phage gradient has been prepared in higher volumes and filled into the plate so that one column has one phage concentration. Bacteria are added to each well of the first row at the same concentration (diluted 100-fold). The bacterial gradient along the columns is prepared in the medium at the respective phage concentration so that it does not change with the bacterial dilution. Even at the highest bacterial concentration, the phage concentration does not change significantly because the bacterial preculture is introduced at a dilution factor of 100.

We have completely rewritten this part of the Methods to make it less confusing and more readable (lines 424-445).

3) I agree with the authors that the phage amplification rate is a very useful measurement of phage efficacy, which might be similar to the bactericidal effect of antibiotics. Yet, as can be seen in many growth curve figures (e.g. Fig. S5), in many cases the repression of bacterial numbers is quite transient and this rebound is obviously an important characteristic for applications like phage therapy as well. It would be good to at least discuss this more.

This is a good point. Indeed, escape mutants often arise. These dynamics are governed by the beneficial mutation rate of the bacteria and growth tradeoffs and are therefore different from the bactericidal effect quantified here. We agree that these resistance parameters are certainly important for therapy. The situation is similar to antibiotics, where it is also not uncommon for resistant mutants to emerge and take over the population during a standard dose-response curve measurement. As with antibiotics, however, it makes sense to clearly separate these two conceptually different issues (measurement of activity, emergence rate of resistance mutants). The present work focuses on the quantification of the phage amplification rate (analogous to the MIC or IC_{50} for an antibiotic). We have simulated the effect of resistance evolution on bacterial growth curves and indeed it does not affect the pre-collapse dynamics. We have actually started another project to quantitatively extract resistance parameters from growth curves, but this is a surprisingly complex problem and beyond the scope of this work.

We have added simulations of resistance evolution (new Suppl. Fig. 4, lines 146-150) and added a discussion of the post-collapse dynamics including resistance evolution (lines 335-342).

Particularly as this might be the reason that final phage yields are not correlated with phage amplification.

The reviewer correctly points out that the final phage yield could be affected by the regrowth of resistant mutants. We have now tested experimentally whether this is the case. We measured the phage concentration at several time points around the collapse and the next day, when regrowth of a resistant mutant has occurred. Importantly, the phage yield after regrowth is only about 1-2 times higher than the phage yield just after the collapse. In

contrast, phage yield varies by ~3 orders of magnitude between different phages. Therefore, we conclude that measuring the phage yield after regrowth does not substantially affect our conclusions.

We have added these results to the manuscript (new Suppl. Fig. 10, lines 488-490, 772-775).

4) Related to the last point, phage yield was used to approximate the phage concentration at the onset of collapse (SI), but phage yield was measured after ON incubation, which is in most cases many hours later than collapse time and it is not quite clear how accurate this approximation is or if it matters.

The reviewer correctly points out that any artifact, such as the one suggested by the same reviewer in point 3, could affect our calculation of the phage amplification factor. Therefore, we calculated the phage amplification factor using either the phage yield as measured after 8 hours or after an overnight incubation. We found that using the phage yield before the collapse substantially underestimated the phage growth rate. However, the phage amplification rate remained within a ~20% range regardless of whether the phage concentration is measured immediately after the collapse, after overnight growth, or at any time in between. These differences are relatively small compared to the differences in phage amplification rate between different phages or different conditions. Therefore, measuring the phage yield after overnight incubation rather than immediately after the collapse considerably simplifies the experiments, without introducing serious artifacts.

We have added these results to the manuscript (new Suppl. Fig. 10, lines 488-490, 772-775).

5) Similarly, the authors approximated p_inf/p_0 by p_stock/p_0 and b0 from bioluminescence for some of the experiments. While they tested the validity of this approximation, it is buried in the Methods. It would be good to include this in a sketch of the method to make it clear, which measurements are necessary (or can be simplified) and how they go into the determination of the amplification rate.

This is a good point. Since the manuscript was submitted, several labs interested in using PHORCE have raised the same issue. Therefore, we have added a detailed description of the various options and requirements for using PHORCE in the Supplementary Information ("Requirements to determine the phage amplification rate").

Out of curiosity: It seems that in Fig. S6c, the approximations lead to better amplification rate estimates with NIT than DOX. Is that coincidence or something to do with how the antibiotics influence phage amplification?

Regarding Suppl. Fig. 6 (Suppl. Fig. 9 in the revised manuscript): This is an interesting observation. While the origin of this effect is not clear, we speculate that it is caused by the fact that DOX has a stronger effect on bioluminescence per cell because it inhibits protein synthesis.

6) The authors use Kendall rank correlation, Pearson correlation and Kruskal-Wallis test for their data but there does not seem to be a statistics section in the Methods, summarizing this data analysis.

Thanks for pointing this out. We have added a new section "Statistics" in the Methods (lines 510-515) where we explain how these analyses were performed.

7) The Methods mention the use of frozen stocks of overnight cultures, which seems a bit unusual, why were not fresh ONCs used?

We have used frozen stocks of overnight cultures for simplicity and because experience in our lab shows that this generally gives nearly identical results and usually increases reproducibility. Note that we also ensure that the bacterial cultures are in a steady state of exponential growth by preculturing for several hours.

We have added an explanation of this point in the Methods (lines 428-432).

8) It is interesting that one phage seemed to have neutral interaction with both antibiotics. Do you have an hypothesis what makes this phage different?

The one phage with a neutral interaction with both antibiotics is indeed remarkable. Unfortunately, it is very difficult to even begin to hypothesize the mechanism behind this neutrality because we selected our phages to represent genetic diversity. As a result, this phage differs from the others in many genes. In addition, the function of most of these genes is unknown. Therefore, we dare not speculate on what makes this phage different from the others. Although this phage is remarkable, we would argue that such an investigation should be preceded by the primary challenge: to understand the general trend followed by most phages (synergism for nitrofurantoin and antagonism for doxycycline). As mentioned in the Discussion, we believe that models of bacterial physiology are a good starting point for this.

Reviewer #2:

The authors set out to build a rigorous mathematical framework and experimental approach to quantify phage activity. They argue that existing experimental methods are error-prone, often ad hoc, and limited by their dependence on difficult-to-acquire phage population dynamic measurements. I agree that the authors are addressing a highly pressing question, as a better quantitative grasp of phage activity is needed to better design phage therapies.

The core of the author's PHORCE method is an expression relating the phage amplification rate, essentially the per-capita growth rate of the phage, to the collapse time of bacterial population. To derive this, the authors define a simple mathematical model of a lytic phage and assume the dynamics are adsorption limited, which they justify by arguing their data is inconsistent with a burst-limited model. In their model, the phage amplification rate is the product of the adsorption rate and the burst size. They show that collapse time with phage Bas04 shows a logarithmic decrease with initial bacterial abundance, consistent with their expression. The authors then perform a series of experiments with Bas04 at varying initial

phage and bacterial concentrations, finding all the collapse times can be explained by fitting a single value of amplification rate.

The authors then apply their method to the BASEL collection of lytic E. coli phages, finding that amplification rates varying across orders of magnitude. As validation, they show that the expected collapse time scaling is generally conserved in this collection. They find a minimal relationship between amplification time and phage yield. Finally, they apply their method to combination treatments of phage and antibiotic. They find that, across multiple phages, trends in phage-antibiotic interaction tend to be determined by the antibiotic, with multiple phages having similar interaction patterns with the same antibiotic.

Overall, I think this manuscript is a worthwhile contribution to the literature. It establishes a more rigorous framework for understanding the dynamical impact of phage on bacteria, akin to MIC's role in understanding antibiotics. However, I have concerns about the generality of the author's mathematical framework and phage amplification metric.

Major comments

1. The authors center their manuscript around the composite phage amplification rate parameter, but do not explore how meaningful this quantity is for predicting phage dynamics beyond collapse time. To what extent does this quantity uniquely define phage dynamics? Can there be substantial difference in the ability of a phage to clear an infection among phage with the same amplification rate?

To address this point, we analyzed the predictions of our model in comparison to the full growth curves for a range of initial bacterial and initial phage concentrations. We found that the model captures the dynamics up to the collapse time using only the phage amplification rate as a fit parameter. Since the phage amplification rate has a monotonic influence on the collapse time, phages with different amplification rates will have different collapse times and vice versa.

The post-collapse dynamics can differ between phages with the same amplification rate, and this part is not captured by the model. The collapse rate is determined by the burst rate, as recently shown (Geng et al., Nat. Com. 2024), and the relapse dynamics are determined by the resistance rate (see response to point 3 of reviewer #1) or, in the case of lysogenic phages, the lysogeny rate (see response to point 3 below).

We have added a comparison between the model and the full growth curves (new Suppl. Fig 3), simulations of relapse dynamics (new Suppl. Fig. 4), and a discussion of post-collapse dynamics (lines 335-342).

Further simulation work exploring the predictive ability of phage amplification rate in a simple mathematical model of phage therapy would improve the manuscript.

It is a great suggestion by the reviewer to compare the phage amplification rate we obtained from laboratory experiments with parameters predicted to be important by models of phage therapy. Since suitable models of phage therapy already exist in the literature, we compared our phage amplification to previous models of phage therapy. In a published model of phage therapy, the control parameter predicting bacterial clearance is the ratio between the phage clearance rate by the body and the product of the phage burst size and the adsorption rate (Leung & Weitz, J. Theor. Biol., 2017). Our work shows how this product of burst size and adsorption rate, which we define as the phage amplification rate, can be readily measured from simple laboratory experiments. The rate at which phages are cleared from the body cannot be measured *in vitro*, and future work should focus on measuring these rates *in vivo* to identify phages with the highest probability of bacterial clearance.

We have added a discussion of the relevance of the phage amplification rate to phage therapy (lines 328-334).

2. The authors show that their model is able to explain the scaling of the collapse time, but is their minimal model able to quantitatively fit the population trajectories of the bacteria? If the model can explain the collapse scaling, but not the trajectory, what is the significance of this deviation? Does it indicate that the amplification rate measured via collapse time is a temporal average? This can be addressed by attempting to fit the model to trajectories and further simulation with modified phage models.

We have now performed these fits (new Suppl. Fig. 3). They show that the model quantitatively captures the dynamics up to the point of the collapse. For a discussion of the post-collapse dynamics, we refer to our response to your point 1 and to point 3 of reviewer #1.

3. The authors consider only a narrow range of possible phage lifestyles, but there is no guarantee that the phage isolated for future phage therapy efforts will obey these constraints. For example, little mention of lysogenic phage is made, how will the accuracy of the estimates be altered if the method is applied to a partially lysogenic phage? The authors should make more extensive simulation of other phage lifestyles to see how their estimates are affected.

Following the reviewer's suggestion, we have now simulated not only lytic but also lysogenic phages. We found that lysogeny does not appreciably affect the collapse dynamics and therefore does not affect the extraction of a phage amplification rate. However, lysogeny does affect the relapse time, as it effectively provides an additional route of resistance via prophage exclusion.

We have added these simulations (lines 821-837, new Suppl. Fig. 4) and a corresponding discussion (lines 147-150) to our manuscript.

Minor comments

1. Given the importance of the model in this manuscript, showing the ODEs in the main text would be worthwhile.

We agree with the reviewer that the model is a central part of our manuscript and we have now put the ODEs in the main text.

2. It is currently unclear how the bacterial growth rate parameter in Eq. 1 is handled in the phage-antibiotic interaction experiments. Did the authors measure bacterial growth rate in the presence of the antibiotic concentration? Or use a growth rate from a no-antibiotic control?

For phage amplification rates in the presence of an antibiotic (e.g., in Fig. 4), we measured r_{bac} in the presence of the antibiotic at the relevant concentration, without any phage.

We have included this explanation in the Methods (lines 533-535).

3. SI Fig 4b may be better suited as a main text figure. Validating that the collapse scaling still works in other species is an extremely important verification step.

We have moved SI Fig. 4b to Fig. 3.

4. Given that the authors only tested two antibiotics, I feel they should soften the generality of their claims on the antibiotics determine phage-antibiotic interactions.

We agree with the reviewer that our claim was perhaps too general in both the abstract and the discussion. We have therefore toned down both sections (lines 24-25, 366-368).

5. Can this method potentially be adapted to study phage-phage interactions?

This is a great suggestion. Indeed, there seems to be potential for our method to provide insight into phage-phage interactions: the model should be able to provide a quantitative null hypothesis of what a bacterial growth curve should look like in the presence of two different phages, based on the individual phage-bacteria growth curves – assuming no phage-phage interactions. Any deviation from the model predictions would indicate interactions. In future work, we plan to extend the model to multi-phage predation and perform experiments to detect phage-phage interactions, which can then be analyzed, e.g. using RNA-seq.

We have added a brief discussion of how the model can be used to detect phage-phage interactions (lines 359-365).

Reviewer #3:

Review "Extreme diversity of phage amplification rates and phage-antibiotic interactions revealed by PHORCE" by Mulla et al.

The manuscript "Extreme diversity of phage amplification rates and phage-antibiotic interactions revealed by PHORCE" by Mulla et al. presents a novel method to directly quantify the bactericidal effect of bacteriophages. Such a method provides important complementary information to traditional phage parameters such as burst size and latent period, and for modeling purposes could eliminate the need to measure these traits, which are elaborate and time-consuming. The manuscript combines experiments and mathematical modeling work. Experiments were performed meticulously and the results seem promising. Since this method would be desirable for enhancing and accelerating quantitative phage research, including fundamental research as well as therapeutic use of phages, we believe that this manuscript can have a great impact in the phage field. We would like to give the authors the following points for consideration. Please mind that you use always line numbers in submitted manuscripts.

Major points:

1. P12 L18, L27, L32, L37 and also in the Discussion, you several times mention "under our conditions" and "adsorption-limited regime". Can you say a few words about the types of phages where this could be different, and how that would impact the approach/results? The finding that the system is adsorption-limited is counter-intuitive. I could only find a brief statement on P13: "While the burst rate may also contribute to the dynamics, assuming adsorption as the sole rate-limiting step already quantitatively explains our results." Still it remains unclear in which cases this might not hold. Could the authors speculate?

The PHORCE model is based on the assumption of adsorption-limited phage dynamics, which we have verified experimentally (Fig. 1c). This assumption holds for all *E. coli* phages we tested (Suppl. Fig. 7) and for a *P. aeruginosa* phage (new Suppl. Fig. 5). However, this is not necessarily true for all growth conditions or all bacteria. For example, for slow-growing bacteria such as pathogenic mycobacterial species, the adsorption rate does not necessarily decrease, but the slower bacterial metabolism is likely to lead to a decrease in the phage burst rate. Therefore, it will be interesting in future work to test the validity of our model for slow-growing bacteria.

We have added a paragraph on the validity of our model in the Discussion (lines 313-319).

2. P13 L42: you mention that "some phage amplification rates were below the detection limit of our technique" - please elaborate, what does that mean? How often does that happen? In the Fig3 caption I found the statement that "slower growing phages did not cause a collapse within the exponential growth regime of the bacteria", but this point should receive more attention in the main text. How common can we expect that to be for other phages, e.g. of faster or slower growing hosts, and/or for other hosts than E. coli? This is important, as it is a caveat that precludes using PHORCE for some bacteria, and this might be very common (we rarely observe collapse events and have worked with many different phages and bacteria). Can the authors provide a directive as to how this issue can be resolved for such cases?

It is true that PHORCE relies on a detectable collapse in liquid growth curves. However, as the reviewer points out, phages often show plaques on bacterial lawns but do not noticeably affect liquid growth curves. In the *E. coli* phage collection we tested, this was the case for 11 out of 69 phages. We speculate that this occurs because these phages have such low amplification rates that phage densities do not reach a critical density to cause a collapse before the bacterial culture reaches stationary phase. For such phages, it will be interesting to develop a similar model to extract phage kinetic parameters from experimental data, using time-resolved plaque growth data instead of growth curves in liquid media.

Slower bacterial growth may result in slower phage dynamics. However, since saturation also occurs at a later time for such bacteria, we expect these effects to largely cancel each other out. Therefore, we do not necessarily expect that collapses will be observed more frequently for fast-growing organisms and a pan-species comparison will be very interesting.

We have added a discussion of the detection limit of our approach (lines 320-327).

This is especially important since the introduction gives off the impression that the method is universally applicable. If only data for E. coli is shown, the paper should strictly be reframed as providing a method that is suitable for E. coli and still needs to be tested on other bacteria, but I realize that would be a pity.

To see if our model is limited to *E. coli*, we also tested a clinical isolate of *Pseudomonas aeruginosa* together with a phage that is used in the Belgian phage therapy center (Queen Astrid Military Hospital). We chose *P. aeruginosa* because it is the target of about half of the phage therapy cases in Belgium. We observed collapses in the liquid medium growth curves for this phage-bacterium combination. Our model accurately captured the dependence of collapse time on initial phage and bacterial concentrations. We extracted a phage amplification rate that is comparable to that of the strongest phages in the BASEL collection. This is plausible because clinical phages such as the one we used in this assay are specifically selected for their high antibacterial activity.

We have added these new data on a phage infecting *P. aeruginosa* (new Suppl. Fig. 5), which support that PHORCE can be used beyond *E. coli*.

3. P14 L22: I'm surprised that "phage yield did not correlate at all with this key quantity (Suppl. Fig. 4d)." Can you give an intuitive explanation? Could you reflect on whether this could be due to the alternative approach of phage particle counting?

We agree that this is an interesting point that deserves more explanation. In fact, we counted the absolute numbers of phages shown in Suppl. Fig. 4d (Suppl. Fig. 7d if the revised manuscript) with plaque assays, so it is not due to an alternative approach of counting phage particles. Our intuitive explanation for why the phage yield does not correlate with the phage amplification rate is that a lower phage yield can be caused by a lower burst size (and therefore lower phage amplification rate), but also by an earlier collapse time (and therefore higher phage amplification rate). In future work, it will be interesting to model and experimentally verify the dependence of the phage yield on microscopic phage kinetics.

We have added a paragraph on this point in the Discussion (lines 305-312).

What about other parameters? In the BASEL phage collection paper they also measured the efficacy of plating (EOP), which might be used as an indication of host range. Maybe you could correlate this with the bactericidal effect to address the "fundamental question" on P17 L21?

The efficacy of plating is typically defined as the number of plaques as observed on a reference host compared to the number of plaques on a host of interest. All the BASEL phages have been isolated on the bacterium we use to test activity. Therefore, there is no reference host other than the host of interest and therefore, by definition, the EOP is 1 for all the phages we studied. We do note that a different EOP effectively changes the burst size, and therefore linearly affects the phage amplification rate.

We have added an explanation of the relationship between the EOP and the phage amplification rate to our manuscript (lines 756-759).

4. P15 L44-45: regarding the statement that "the phage yield decreases similarly with increasing concentration for both antibiotics (Fig. 4d)" and "In contrast to the phage amplification rate, the yield is not markedly different between the two drugs" (Fig4 caption). It looks like the yield drops for NIT, how significant is that? Would you discuss what could be an explanation (maybe quicker release with lower burst size)? Also, the Y axis of Fig 4d is inconveniently cut at the low end, please fix this. While in Suppl fig 6b, there is no general trend between the interaction scores, the specific cases represented by outliers on the X and Y axes may still be interesting to note and/or investigate further?

Indeed, for this specific phage, we find that the yield decreases significantly as a function of the nitrofurantoin concentration. In terms of mechanism, this essentially boils down to point 3 above: it would certainly be interesting to understand the dependence of the phage yield on microscopic phage parameters. Once we understand this relationship, we can begin to understand the dependence of phage yield on specific antibiotics for specific phages, which we agree is a worthwhile avenue for future research.

We have added a perspective on understanding antibiotic-dependent phage yield to our discussion (lines 309-312). Furthermore, we have updated the y-axis of Fig. 4d.

5. P16 L2-5: "Together with the lack of correlation between phage amplification rate and phage yield across the entire BASEL collection (Suppl. Fig. 4d), these results show that the phage amplification rate is a distinct property of phages that is largely independent of the commonly used phage yield." From this statement, the parameter should be termed differently, e.g. "bactericidal effect" or "virulence". This is because the "phage amplification rate" is intuitively connected to phage growth, but this parameter actually reflects the ability of the phage to inhibit bacterial growth.

The phage amplification rate, as we define it, microscopically reflects the rate at which phages amplify. It is correct that this single parameter explains the macroscopic bactericidal

effect. Therefore, we believe that both terms are correct in principle. However, there have been previous attempts to capture the bactericidal phage effects from growth curves, such as the “virulence index” (Storms et al., Phage, 2020). These measures are purely phenomenological and have no microscopic mechanistic basis. Indeed, the phage amplification rate as defined by us captures the macroscopic behavior more effectively than previous phenomenological approaches, but we want to emphasize its microscopic basis as it is qualitatively novel – and thus prefer to keep the term “phage amplification rate.”

6. P16 L21: "directly compared across different phages, hosts, and growth conditions" but two lines later you say: "hard to standardize across experiments". How stable is the phage amplification rate for different culturing conditions? If we want to compare phages from different studies and systems, experimental conditions are bound to differ, so this will impact conclusions and interpretation of more complex situations. Please address this potential caveat in sufficient detail.

The phage amplification rate is indeed dependent on culture conditions. For example, we see a strong dependence on the antibiotic concentration, and we expect similarly strong differences between, e.g., rich and minimal media or at different temperatures. Such a dependence on growth conditions is a more general problem; for example, it is also known to occur for the minimal inhibitory concentration of an antibiotic, which can be strongly influenced by growth conditions (e.g., Angermayr et al., Mol Syst Biol, 2022). It will be interesting to see which culture conditions best predict *in vivo* phage activity using preclinical infection models as a first step towards mimicking patient conditions. It will also be interesting to perform cross-laboratory validation of reproducibility under similar culture conditions.

We have added a discussion on the condition dependency (lines 297-304).

7. P18 L32, P19 L12: I really like that the methods for "Two-dimensional gradient of initial phage and bacterial concentration" and "Antibiotic gradients" are thoroughly described, but they are quite difficult to follow and it is difficult to visualize the experiment from the text alone. For example, "Subsequently, a gradient of phage titers was established in a 12-column deep well plate using a 1:2 serial dilution procedure, resulting in 6 ml per phage concentration." It is hard to understand how the 6 ml per phage concentration comes about. An addition of figures or organizing the steps would make it easier to understand.

We regret that this part of the Methods was difficult to follow. Reviewer #1 raised the same issue in their point 3. We have thoroughly revised and simplified this text and hope that both reviewers will find it easier to follow.

Minor points:

8. Please remove the word "Extreme" from the title, as this is subjective and unnecessary.

We observe a diversity of phage amplification rates spanning about four orders of magnitude, which is larger than the difference between very slow and very fast growing

bacteria. Notably, we find this diversity even between phages infecting the same bacterial strain. Therefore, we prefer to keep the word “extreme” in the title. However, we can remove it from the title if this is the consensus view of the editor and reviewers.

9. P10 L9-11: Rephrase "The resulting phage amplification rate captures the bactericidal effect independent of initial phage and bacterial population sizes and across different growth conditions" based on your results.

We have rephrased the sentence (“We found that the resulting…”, lines 20-22).

10. P11 L15: "Burst rate" should be changed to "Latent period", as this term is more commonly used to define the time between adsorption to the release of new progeny.

Thanks for pointing this out; we have now changed the burst rate into the latent period throughout the manuscript.

11. P11 L15-17: it is suggested that Fig 1a is based on time-resolved measurement of phage concentrations, but the Y axis reflects bacterial abundance.

This was indeed confusing; we removed the reference to Fig. 1a.

12. P11 L27: rephrase "phage amplification rate in dependence of the bacterial concentration" (in dependence is unclear, maybe "depending on")

We rephrased it to “the bacterial concentration-dependent phage amplification rate.”

13. P11 L34: Define BASEL as BActeriophage SElection for your Laboratory.

We have added the full form of BASEL (*BActeriophage SElection for your Laboratory*) to the manuscript.

14. P11 L41 I think the start of the Results section could do with a few introductory sentences. For example, with the term "dynamics of a phage population" do you mean the changes in abundance of the phages over time? I thought you rather "aimed to quantitatively understand" phage virulence parameters? Please clarify. Also on Pg 4 L17.

We agree with the reviewer that the wording at the beginning of the Results section is unclear. We have now changed it to quantitatively understanding “the bactericidal activity of phages” in a growing bacterial population.

15. P12 L1: E. coli nor phages are generally bioluminescent. A few more words on the experimental design would be helpful. Additionally, please provide further clarification on how you ensured that all cells were carrying the plasmid throughout the experiment, as plasmid loss over time is known to occur.

We have added to the main text that the *E. coli* bacteria carry a plasmid encoding luciferase genes. We have previously shown that this plasmid is stably maintained in the bacteria for many generations even in the absence of the selective antibiotic (Suppl. Fig. 7c in Kavčič et al., Nat. Com., 2020). We have now added this information to the Methods (lines 400-401).

16. P12, L5, it is mentioned that the collapse time was measured using bioluminescence. Typically, optical density (OD) is the standard method for such measurements, and the correlation between bioluminescence and OD measurements was initially unclear to this reviewer. I recommend including the current Supplementary Figure 1b within the main Figure 1, as it effectively demonstrates the strong correlation between these two measurement methods. This would provide reassurance about the validity of using bioluminescence as a proxy for optical density.

As the comparison between OD and bioluminescence is a technicality, we prefer to keep it in the Supplementary Figure. We agree with the reviewer though that it is an important one, therefore we have added a reference to the comparison in the caption of Fig. 1 (lines 702-703).

17. P12 L21-22: please clarify "absolute concentration" of what?

We have revised the text to clarify that this is about the absolute concentrations of bacteria and phages (lines 101-103).

18. P12 L30: fig 2c is mentioned in the text before 2a and 2b, move up in figure, or make a separate figure?

We have revised the figure to make sure it follows the order of the text.

19. P13 L43: the sentence "To put this diversity into perspective..." - please add the actual values, and add some refs.

We have now added references and the actual values of the growth rates (lines 178-179).

20. P14 L9: "such as genome size or primary/terminal receptor" where is this data shown? Please refer to a table or figure.

We have now added a table with all the phage metadata such as genome size and primary/terminal receptor (new Supplementary Table 1).

21. P15 L35-36: "phages that showed any interaction at all generally synergized with nitrofurantoin but were antagonized by doxycycline" were these the same phages? A Supplementary Table with all details about the phages in all experiments (not just the antibiotics) would be valuable.

These were indeed the same phages, and Fig. 4e shows that there is no correlation in the interaction score between the two drugs. The phage names are listed in the Figure legend. We have now added a table with the phage metadata (new Supplementary Table 1).

22. P17 L31-32: Please rephrase "phage amplification laws" as it is unclear what you are talking about.

The phage amplification laws draw an analogy to bacterial growth laws, which is a concept in a subfield of bacterial physiology. However, we realize that this will only create confusion for people outside of this subfield. Therefore, we have decided to remove this sentence from the manuscript.

23. P18 L22-26: Can you briefly explain why the spent medium is used for phage dilution, and not fresh medium? Also please describe how the spent medium is made.

We apologize for the confusion. We did not use spent medium in any of the experiments described and have now removed the spent medium part from the Methods section.

24. P18 L34, P19 L14, L18, L20: In the methodology section, the dilution levels of bacteria and phage are mentioned, while in the figures like Fig 2a-b, Fig 3b, the concentrations are provided. To ensure consistency and enhance the clarity of the methodology, could you please include both the dilutions and the corresponding concentrations of bacteria and phage directly in the methodology section.

We have added these bacterial and phage concentrations to the Methods (lines 435-438). We have also added a column to the new Supplementary Table that lists the stock concentration of each phage.

25. P18 L35: Define ONC as bacterial overnight culture.

We have removed the abbreviation and now only use the full form.

26. P19 L38: "Phage purification" From the description this step is to extract phage particles from bacterial culture. At first I thought "phage purification" referred to the method to purify a single phage strain by culturing from a sample, involving multiple plaque assay steps. So maybe an additional context can help to mitigate this? For example "Phage purification from bacterial culture / Phage isolation".

We have changed the heading to “Phage purification from bacterial culture.”

27. P20 L27: "Drop assay for stock concentrations of phage samples" This could be named "Spot assay" to make it easier to understand. The term drop assay is not commonly used.

Thanks for pointing this out. We have changed the title accordingly.

28. P29 Fig 3c: could you explain where the unit of the X axis comes from, shouldn't this be something like particles per hour?

The phage amplification rate is obtained by multiplying the burst size by the adsorption rate. The burst size is a ratio and therefore a dimensionless number. The unit of the adsorption rate is the inverse bacterial concentration (ml) per unit of time (h). Since the number of bacteria is not an SI unit, it is common not to introduce a unit ("bacteria") for it, but just to leave it as a number (see, e.g., Leung & Weitz, J. Theor. Biol., 2017). Thus, we end up with the unit ml/h as shown in the figure.

29. Fig 3d: it looks like Autographiviridae may be significantly higher than the others, is it? Did you correct for multiple testing? This would be appropriate.

We have checked for the various phage families, and while the Autographiviridae do have a higher average phage amplification rate, this is not statistically significant even without correcting for multiple testing. The reviewer correctly points out the importance of multiple testing, which reduces significance values when multiple groups are evaluated. We used a Bonferroni correction for this purpose. With this correction, the p-value for Vequintaviruses still supports a highly significant lower phage amplification rate. We have described this new correction in the figure legend (lines 726-727) and in the Methods section (lines 511-512).

30. P30 in Fig 4e you use a P value cutoff of 0.05 ("The dashed line is at p=0.05"). If you want to use a cutoff, it should be corrected for multiple testing.

In examining the general distributions between the two categories (neutral, antagonistic, or synergistic) in Fig. 4e (now Fig. 4d), we are not focusing on specific phages, but on overall patterns. While we agree that multiple testing corrections are important when testing specific hypotheses about individual phages (e.g., differences in amplification rates as discussed in point 29 above), applying such corrections here would lower the significance of all p-values. This could obscure the true differences in the distributions and artificially inflate the proportion of neutral interactions as more phages are tested, masking the actual distinction between the effects of the two drugs. Therefore, we have chosen not to apply multiple testing corrections and instead report on the overall distribution of interactions, which is central to our analysis.

31. Comparing Fig 4e and 4f, it seems like the axis labels in 4f are reversed? Note that it is difficult to identify the different markers, especially when they overlap. Maybe they can be made a bit bigger, and more different.

The reviewer is right, we apologize for the reversed axis labels in Fig. 4f. We have now corrected this error (now Fig. 4e). We have also made the markers larger and some different to improve readability.

32. Fig 4f caption: please put the marker legend in the figure.

We have tried putting the marker legend in Fig. 4f (now Fig. 4e) however this would only fit if we made the marker legend so small that it became unreadable. Therefore, we have put the marker legend in the caption.

33. Several times you mention "bootstrapping" the growth curves, but it is unclear what that means/how this is done. Do you mean replicates? In the fig1 and fig4 captions, could/should you mention how many times (1000x)?

Bootstrapping involves randomly resampling the original data many times and analyzing each resampled set. It can be used to estimate the error of experimental observables or derived quantities. We have added a brief description to the Statistics section in the Methods and now refer to a more detailed description elsewhere (lines 513-515).
