# Supplementary material for: Extreme diversity of phage amplification rates and phage–antibiotic interactions revealed by PHORCE
Source: PLoS Biol. 2025 Apr 8;23(4):e3003065. doi: 10.1371/journal.pbio.3003065 (PMC12013923; doi:10.1371/journal.pbio.3003065)
Supplement: Attachment — Submitted filename: PHORCE_point_by_point_response.docx [file pbio.3003065.s017.docx]

**Point-by-point response**

**We thank the editor and reviewers for their input. Below we address all comments from both the editor and the reviewers.**

a) After discussing within the team and with the Academic Editor, we were thinking that the study could fit well as a Methods and Resources type of article. If you agree that the focus should be on the application of PHORCE, we would encourage to change the type of format during re-submission

**We appreciate the suggestion and agree to change the format type to Methods and Resources. Note that we have not been able to change the article type in the submission system; hopefully this can be fixed later.**

b) You may be aware of the PLOS Data Policy, which requires that all data be made available without restriction: <http://journals.plos.org/plosbiology/s/data-availability>. For more information, please also see this editorial: <http://dx.doi.org/10.1371/journal.pbio.1001797>
Please supply the numerical values either in the a supplementary file or as a permanent DOI’d deposition for the following figures: Figure 1bc, 2bd, 3bcde, 4a-e, S1ab, S2ab, S3, S4, S5abc, S6, S7a-d, S8, S9abc, S10abc*

NOTE: the numerical data provided should include all replicates AND the way in which the plotted mean and errors were derived (it should not present only the mean/average values).
*If some are just examples of the model, please disregard the request

**We now provide all numerical experimental data for all figures mentioned as a supplementary data sheet.**

c) Please cite the location of the data clearly in all relevant main and supplementary Figure legends, e.g. “The data underlying this Figure can be found in S1 Data” or “The data underlying this Figure can be found in <https://doi.org/10.5281/zenodo.XXXXX%E2%80%9D>

**All main and supplementary Figure legends now indicate where the data can be found.**

d) We do not have a word limit, even for Short Reports. Could you please move the section named "Model of phage amplification kinetics" to the Materials and Methods in the main text? This would allow readers an easier access to the model.

**We have moved the section on the details of the model from the Supplementary Information to the Materials and Methods section.**

e) Please ensure that your Data Statement in the submission system accurately describes where your data can be found and is in final format, as it will be published as written there.

**We have added a description of where the data can be found to the data statement.**

f) Per journal policy, if you have generated any custom code during the course of this investigation, please make it available without restrictions upon publication. Please ensure that the code is sufficiently well documented and reusable, and that your Data Statement in the Editorial Manager submission system accurately describes where your code can be found.
Please note that we cannot accept sole deposition of code in GitHub, as this could be changed after publication. However, you can archive this version of your publicly available GitHub code to Zenodo. Once you do this, it will generate a DOI number, which you will need to provide in the Data Accessibility Statement (you are welcome to also provide the GitHub access information). See the process for doing this here: <https://docs.github.com/en/repositories/archiving-a-github-repository/referencing-and-citing-content>

**We have uploaded the data analysis code to Zenodo and have added a reference to the DOI in the Methods section where the code is explained.**

------------------------------------------------------------------------
REVIEWERS' COMMENTS
------------------------------------------------------------------------
Reviewer #2:
I apologize for the delays in my review. I am impressed by the revisions
that the authors have made, and believe that the new manuscript is suitable
for publication in PLoS Biology.

**We thank the reviewer for their kind words.**
------------------------------------------------------------------------
Reviewer #3:
We would like to thank the authors for responding to and addressing most of our previous comments. Overall, this work presents a novel and interesting concept for a new parameter that captures bacteriophage biology, and the effort from the authors to make this protocol accessible to other researchers is evident. However, we still find that the title and abstract suggest that the method is universally applicable, while it is not. We would thus like to offer some further suggestions to be more upfront about the caveats of this work. We feel that this will not detract from the impact, while avoiding potential disappointments for researchers who are not working on fast-growing hosts or adsorption-limited phages.

**We appreciate the reviewer's positive feedback and thoughtful comments. Below, we address the specific points raised to further clarify our work.**

While the addition of Pseudomonas aeruginosa provided valuable insights, it does not address the lack of universal applicability of the PHORCE method. Both E. coli and P. aeruginosa are fast-growing, gram-negative opportunists. To address the issue and move forward, we suggest replacing the sentence in the Abstract: "We found that the resulting phage amplification rate captures the bactericidal effect independent of initial phage and bacterial population sizes and across different growth conditions." by a sentence along the lines of "We found that the resulting phage amplification rate captures the bactericidal effect independent of initial phage and bacterial population sizes, and works well for fast-growing hosts and adsorption-limited
phages." Removing the last few words of the original sentence seems justified, since apart from the addition of antibiotics, the study does not address different growth conditions.

**We agree that it is important to be clear about any limitations of the method. We have therefore changed the sentence in the abstract as suggested by the reviewer.**

Please also remove the speculation in line 299 that "differences between, e.g., rich and minimal media or at different temperatures" strongly impact phage amplification, as this seems preliminary.

**We agree that as a claim, this sentence would be preliminary. To clarify that this line in the discussion is a speculative, we have changed the beginning of the sentence from “we also expect” to “we speculate”. We also removed the word ‘strongly’ in “strongly affected.”**

Re: Reviewer #3 point 2
The authors speculate about the reason that 11/69 E. coli phages do not noticeably affect liquid growth curves, hypothesizing that these phages have such low amplification rates that phage densities do not reach a critical density to cause a collapse before the bacterial culture reaches stationary phase. However, a lack of observable collapse is not always attributed to low amplification rate, but could also reflect a stable coexistence between phage and host (doi: 10.1038/s41467-018-07225-7, 10.1186/s40168-021-01036-7). Please discuss this.

**We agree that it is good to point out that others have also observed phages that can form plaques but show no collapse in liquid media, and that alternative explanations have been proposed. We have therefore added the following sentence to the Discussion: “Phages showing plaques on solid agar but no collapse in liquid media have previously been attributed to potential different infection states occurring in liquid, and it would be interesting to experimentally verify the existence of such states.” In this sentence, we cite doi: 10.1038/s41467-018-07225-7 (Ref. 28 in the manuscript).**

Re: Reviewer #3 point 5
We agree that the observed phenomenon, i.e. the inflection point in the growth curve which is used for calculating the phage amplification rate using PHORCE, most likely stems from phages that are amplifying and killing bacteria. However, there are many examples of situations where phage amplification does not result in a drop in the growth curve (see refs above). In such cases, PHORCE is not suitable for calculating phage amplification. We would like to ask the authors again to reconsider the term "phage amplification rate" to avoid confusion, could they replace by something that includes the killing aspect?

**We acknowledge that PHORCE is not suitable for calculating phage amplification in cases where amplification does not lead to a measurable decline in the growth curve. However, this detectability threshold does not change the fundamental nature of the measurement itself. The term *phage amplification rate* accurately reflects the biological process captured by our model, which inherently links amplification to bacterial killing. As such, we maintain that this terminology remains the most accurate descriptor for the metric derived from PHORCE.**

Re: Reviewer #3 point 8
Regarding the word "extreme": the effect spans >3 orders of magnitude (not 4 as stated in the rebuttal). It seems appropriate to be specific, both in the title and Figure 3 caption, so e.g. "Phage amplification rates and phage-antibiotic interactions span three orders of magnitude, as revealed by PHORCE".

**We appreciate the reviewer’s suggestion and acknowledge the importance of precision in scientific communication. However, we prefer to retain the word *extreme* in the title, as it succinctly captures the significant diversity observed in our data while remaining reasonable and interpretable. As noted in our previous rebuttal, the observed diversity exceeds three orders of magnitude, which we believe justifies the use of *extreme*. The exact numerical range is explicitly stated in the abstract to ensure clarity for the reader.**

**In addition, we find the proposed alternative title problematic, as the phrase “spans three orders of magnitude” could be misleading. While this range applies to phage amplification rates, it does not describe the phage-antibiotic interactions, which could lead to confusion.**

**As a compromise, if the editor agrees that keeping *extreme* is not acceptable, we propose either replacing it with *vast*—which conveys a similar meaning while being slightly less strong—or removing the word altogether without replacing it with “more than three orders of magnitude.” However, we believe that removing a qualitative descriptor would make the title less engaging and potentially reduce its impact.**

**We hope this explanation clarifies our thinking and are open to further discussion.**

The manuscript (and rebuttal) now several times mentions "microscopic phage kinetics", but we find this word confusing, as there is no microscope involved. Please rephrase.

**We understand that the use of the word “microscopic” can be confusing. We have removed it from the manuscript.**
